# On Functional and Semi-Organic Diseases of the Heart

**Published:** 1872-08

**Authors:** John E. Lockbridge

**Affiliations:** Augusta County, Virginia


THE GEORGIA
MEDICAL COMPANION:
A MONTHLY ADVISER.

VOL. 2. ATLANTA, GA., AUGUST, 1872. No. 8.

### PART I.

### Original Communications and Special Selections.

### ON FUNCTIONAL AND SEMI-ORGANIC DISEASES OF THE HEART.

BY JOHN E. LOCKBRIDGE, M. D., OF AUGUSTA COUNTY, VIRGINIA.

Of all the maladies to which the human flesh is heir, there are none, perhaps, more perplexing to the young practitioner of medicine than affections of the heart; and this is especially true in respect of the diagnosis between functional or semi-organic and organic diseases of the organ. In truth, no practitioner can be an expert in the diagnosis or treatment of these complaints, unles he has a profound knowledge of ausculation and percussion; as well as of physiology, pathology, natural philosophy, and acoustics. The writer does not profess to be an adept in a single one of these sciences.

The object of this paper is merely to chronicle some observations on the functional and semi-organic diseases—without discussing at all the organic diseases of the heart—and this, too, in a very practical and cursory manner; and also to give a very brief sketch of some cases which have occurred to him in practice, somewhat illustrative of the several conditions herein discussed.

One decade in the dark past of medicine every cardiac murmur, with the single exception of the so-called anæmic or hygræmic murmur, was regarded as a harbinger of an almost necessarily fatal issue—indeed, it was generally looked upon as a death knell.

But the experience and investigations of the past ten years have wrought a wonderfully wholesome change in the minds of practitioners in regard to a great many of these murmurs. Murmurs that once gave rise to the most fearful forebodings, are now known to be of very trifling importance. For this change in our prognosis we are indebted to no one more than to Prof. DaCosta, of Philadelphia.

The following are some of the conditions in which we hear these murmurs, viz: anemia, chlorosis, chorea, hysteria, spinal irritation, fatty degeneration of the cardiac parietes, irritable heart, paralysis &c., &c.

It is clearly my opinion that two pathological conditions, and only two, give rise to all these murmurs, viz: hypertension-contraction-spasm of the columnæ carnæ, and hypotension-flacidity-paralysis of the same. It is only necessary for me to give the merest sketch of the structure and functions of the fleshy columns, tendinous chords (*chordæ tendinæ*), and their attached valves. The fleshy columns are the muscles of the valves, and the tendinous cords are the tendons of these muscles, and are also attached by the other extremity to the valves. It is very readily understood in what manner the valves are opened and closed by a successive contraction and relaxation of these fleshy columns in their strictly physiological condition—the columns and tendons being exactly suited in length to reach the auriculo-ventricular foramina, and the valves being precisely suited in size to their openings.

Now, it is perfectly apparent that these columns and cords cannot perfectly subtend the distance to the foramina if the former be in a condition either of excessive or diminished tension; for in the latter case the valve will be allowed to recede into the corresponding auricle, and in the former condition, it will be arrested by the cords before it reaches the auriculo-ventricular foramen.

In both of these conditions there will be a regurgitation of blood from the ventricle to the auricle, through the imperfectly closed valve, and the resultant cardiac murmur. So it is very plain that in case we have either excessive condensation, or spasm, tonic or clonic of the fleshy columns; or, on the other hand, excessive relaxation or paralysis of the same, we have a murmur. It will be seen from the foregoing remarks that the immediate cause of systolic regurgitant murmurs *alone* have been accounted for by the opposite two lesions in question. And, in truth, from a practical stand-point, very little need be said in regard to diastolic functional mumurs: for they are seldom heard, and never, to my recollection, by me. Williams is cited twenty years ago as saying that five-sixths of the murmurs that he had met with were not only regurgitant, but mitral regurgitant; and that, too, at a time when the scope of the bellows murmur was limited almost exclusively to organic diseases of the organ.

I do not wish to be understood as attributing the cause of these functional murmurs solely to increased or diminished tension of these fleshy columns; for the cause is doubtless composite to some extent, the same pathological conditions extending to, and influencing and controlling the action of the valves themselves, as well as the cardiac parietes. I believe, however, that the fleshy columns act, by far, the most prominent part in the production of the abnormal sounds.

So far as my observation goes—for I have only been paying particular attention to these abnormal sounds for the last five or or six years—the murmur has invariably been mitral regurgitant —that is, systolic, below, and to the left. And how does my philosophy accord with the anatomy and physiology of the heart? The parietes of the left ventricle are three times as thick as those of the right; the columnæ carnæ are much more fully developed, and this is especially true of the *columnœ papillares*, of which there are only two—one of which is attached to each mitral valve at one extremity, by means of its tendinous cord; and by its other extremity to the walls of the ventricle. Now, it is very evident to me that if a part or organ is more strongly developed than another organ, in order that it may be adequate to the performance of a more arduous function, it will surely follow that a failure of this part to perform its duty, or a disposition to take upon itself a work of supererogation, will be attended with more manifest results. And this is precisely true of the right and left sides of the heart; as well as of its systolic and diastolic action: and we would surely expect the mitral regurgitant murmur much more frequently from the foregoing pathological conditions.

I have already alluded, incidentally, to the most frequent remote causes, or *causæ causarum*, of these functional murmurs. Of the predisposing causes, the most frequent, according to my observation, are spinal irritation, irritable heart, paralysis, chorea, dyspepsia, epilepsy, a general tendency to spasm, hysteria, the excessive use of tobacco, old age; the last stage of typhoid fever, and other diseases characterized by great depression of the physical organism; and last but by no means, the least frequent cause, the so-called hygræmic condition of the blood. For, whilst I admit that "blood runs thicker than water," yet I am more disposed to attribute the murmur to imperfections of the channel than to the character of the flowing liquid. In respect to the murmurs so often heard in the last stages of typhoid fever, as well as the irregularity of the heart's action, these are more likely to be due to the want of cardiac tension, than softening of the parietes—to which cause some writers have assigned their production. Although these causes are as diversified as possible, yet they are as reconcilable as possible, when we remember that, although the heart is capable of contracting and dilating even when removed from the body, and totally severed from all nervous influence; yet its action is greatly influenced by every kind of influence and emotion.*

I know of no additional light that I can throw on the differential diagnosis of functional and organic diseases of the heart. An opinion is only to be formed by experience, and a very careful and minute investigation of each particular case, together with a judicious appreciation of the data thereby elicited. In cases that turned out to be purely functional, I have heard the *bruit de souffle*, as well as the *bruit de diable* and *bruit de scie*. I have thought,

---

*Through the catenation of the cerebro-spinal, par vagum, and ganglionic systems of nerves.

however, that when the objective symptoms are very palpable, and the physical signs very loudly developed, an opinion might be given in favor of functional disease—and especially so, in the absence of hypertrophy or dilatation, or when the area of cardiac dullness is but very slightly increased.

In regard to the prognosis, I have no hesitancy in saying that it is as favorable as possible, except in such cases as those in which paralysis, old age, fatty degeneration of the heart, the last stages of typhoid fever, and other low diseases stand as the remote cause. In truth, since I have been noticing these troubles particularly, every case has fully recovered except a youth who was dying of typhoid fever, and an old lady, whose case is still under consideration, and whose I shall report below. I have styled some of the cases semi-organic, for the reason that, although there is no histological change in the interior structure of the heart, as respects volume or appearance, or heterotopia of tissue; yet, in one of the pathological conditions which I have assumed to exist, there is diminished tension in the fleshy columns, and doubtless in the cardiac parietes; and this in some cases is capable of superinducing hypertrophy with attenuation of the walls, or eccentric hypertrophy. Although I have found the hypertrophy of dilatation to exist in some cases to an appreciable extent, yet the heart ultimately returned to its normal proportions. Hence, I conclude that although the same causes exists by which true and false hypertrophy are produced in a heart which is organically diseased, viz: overdistention of the chambers, and an increased amount of work; and in consequence a compensative and conservative hypertrophy; or, on the other hand, a deadly dilatation and attenuation of the walls: yet, whatever change may have been wrought is capable of being perfectly rectified. This radical difference in the behavior of the two conditions is doubtless due to the kakoplastic metamorphoses that have taken place in the valves and orifices having extended to the cardiac parietes—whether these changes be of rheumatic origin, or softening, or fatty degeneration—and as a legitimate result leading to conpensative hypertrophy, or the more unfortunate result, eccentric hypertrophy with attenuation of the walls; both of which conditions are perhaps incurable, and incapable of being rendered normal in their proportions.

As respects the treatment of these murmurs, I have nothing to add, as it consists in merely rectifying the particular fault of system which stands as the predisposing cause of the case that may be under consideration, together with the judicious combination of such remedies as are known to have a specific action upon the heart and nervous centres. As I have already extended this paper beyond the limit compatible with the plan of this (our) journal, I will conclude by appending a very short sketch of a few cases illustrative of the symptoms and treatment of the conditions under consideration.

Case 1. 1865. Mrs. ——, aged 29, married 11 years, without children; previous health bad; has been the subject of frequent and very severe attacks of inflammatory rheumatism, the last of which was in February, 1857; ever since the establishment of the catamenia has had amenorrhœa, and annual, and sometimes semi-annual attacks of excessive metrorrhagia; had one of these a few months ago; rather anæmic; weight about 130 pounds; whilst I had my ear near the chest, I accidentally heard a cardiac murmur, which, upon examination, proved to be a mitral regurgitant murmur. It could be heard several inches from the chest, and was exceedingly loud. There seemed to be an appreciable increase in the area of precordial dullness. My friend, the late Dr. C. R. Harris, saw this case with me, and ascribed the murmur to anemia. I was disposed to fear that the trouble was the result of rheumatism. For over two years the treatment was tonic and expectant, with no change in the murmur, and very little in the general condition of the patient; and but little hope of the favorable termination of the case. In the spring of 1868 the symptoms became very much aggravated. The patient, on two or three occasions, fell almost lifeless upon the floor on rising in the morning; there were attacks of syncope; a good deal of pain and great distress were now experienced about the heart. Upon examination the murmur existed unchanged; there was great tenderness on pressure of the spine between the scapulæ, and firm pressure greatly aggravated the heart symptoms. A more heroic treatment was now instituted. I prescribed the iodide and bromide of potassium 3 times a day, with a succession of blisters to the spine, and an occasional blister over the region of the heart. The symptoms began at once to improve; the patient's comfort was greatly increased, and the murmur began to grow fainter. By the first of July the patient was able to take a trip; she traveled over the Western States of the Union until the middle of September. Whilst in Illinois she had an attack of intermittent fever, for the cure of which she took large doses of quinia. She returned home about the 15th of September cured, or nearly so, and has been in perfect health ever since. There is no murmur or other evidence of cardiac disease; and the patient now weighs 165 pounds. Before leaving this case, it is proper that I should mention that after her return in 1868, she was threatened with another attack of metrorhagia, for the control of which large doses of ergot were administered, which produced excessive uterine contraction and pain, with the expulsion from the uterine cavity of a cellular polypus the size of the end of the thumb; after which the hemorrhage ceased. I removed this polypus some weeks afterwards, and she has not had a hemorrhage since, and the catamenia have been regular. This was doubtless a case in which spinal irritation and spasm of the fleshy columns produced the murmur.

Case 2. In the summer of 1868 I examined Mr. ——, aged about 30 years, with the late Dr. C. R. Harris. I was told that he had been a subject of heart disease for the last 8 or 10 years. He was reduced to an almost extreme degree of emaciation; his appearance was haggard in the extreme, and, in short, the objective symptoms were in the very highest degree typical of organic disease of the heart. His death was regarded by his friends and physicians as being near at hand, and only a question of time. He was the subject of very frequent attacks of something like syncope, from some of which he had barely recovered. His physicians, some of them not only the best in his county, but equal to any in the State, had long since abandoned the case as hopeless. Upon examining him physically I found no murmur to exist at that time, nor was there any appreciable increase in the area of precordial dullness; there were no physical signs indicating disease of the heart at this time, except some excitement of the organ. The patient was very calm at the time of examination, which was in the morning. Doubtless, there had been murmus and other cardiac disease which led his physicians to diagnose organic disease of the organ. I found dullness on percussion over the left infra-clavicular region, with broncophony and bronchial respiration. There was an extreme degree of tenderness of the spine about the lower cervical and upper dorsal vertebræ. We diagnosed the case as spinal irritation, and the trouble about the apex of the left lung as being due to the result of passive congestion. To the amazement of the patient and friends, we gave as favorable a prognosis as possible. We prescribed 25 drops of the syrup of the iodide of iron 3 times a day, to be washed down with a liberal amout of whisky; and directed that the spine be kept constantly pustulated with croton oil. The treatment was faithfully carried out, and he began to improve immediately. As he resided in an adjoining county, I did not see him again until the latter part of the year 1869, when he again visited his relatives in my neighborhood, in company with his blooming bride. He was then in the enjoyment of most excellent health. More than a year later he was a father and a widower—his wife having unfortunately died in her accouchment. This gentleman is now in very active business in the city of Richmond, Va., and he is, perhaps, as fine a specimen of manly vigor, as well as moral and Christian excellence, as the city affords.* This was a case of spinal irritation, producing an irritable heart, which had rioted in excess until the point of complete exhaustion had arrived.

Case 3. On May 27th, 1871, I was halted by a man on the road-side, who had followed me on foot some 8 or 10 miles, wishing to consult me about some heart trouble with which he was suffering. He was about 50 years of age, and presented a very care-worn and haggard appearance, and, withall, seemed to be very much depressed in spirits. I had treated this man in the preceding January for functional paralysis; it was general in its nature, and was evidently the result of exposure in extremely wet

* This gentleman is over six feet high, and increased in weight from 130 to 175 or 180 pounds.

and cold weather without being protected with adequate clothing. In a few days the paralysis had almost disappeared, and he resumed his work as usual, and I saw nothing more of him until the above date, May 27th.

He complained of great oppression about the chest, with some difficulty of breathing; he informed me that on rising in the morning he felt like fainting, and had actually fallen on the floor on several occasions. On putting my ear to his chest outside of his vest I at once detected a startlingly distinct murmur of the heart. On closer examination it was very readily ascertained to be a mitral regurgitant murmur. There remained no outward manifestation of paralysis, except an inability to perform perfectly the act of expiration. There was very marked exopthalmos, especially so of the left eye. He complained of being very weak. I told him that he would probably get well, and prescribed the following pills: R.—Iron by hydrogen, dr ii; Pulv. digitalis; Ext. nux vomica, aa grs xv; Water, q s.—M. Ft. pills No. 30. Sig. One to be taken three times a day.

June 13, I visited this patient at his home, and found him very much improved in every particular; murmur less distinct, but still plainly audible; has had no attack of syncope since I saw him, and is quite comfortable. Continue treatment.

June 24.—Saw the patient again, and found him almost entirely relieved; murmur has entirely disappeared, and he feels quite comfortable with the exception of some degree of weakness. Continue treatment; discharged. I saw this man some weeks afterwards on the road to see his brother, on foot, and he seemed to be quite well and active.

This was doubtless a case of cardiac paralysis—the paralysis, for the most part being confined to the fleshy columns. This man became insane in the latter part of the year 1871, was confined in an asylum, and has since died.

Case 5—May, 1872. I was called to see Mrs. R., aged 88 years. She has had great difficulty of breathing for the last 36 hours, with inability to lie in the horizontal position; slept none last night; she was sitting in her chair without any very marked trouble in breathing; but great trouble whenever she attempts to lie down; complains of great weakness and loss of appetite; pulse about 93 to the minute, and very weak and intermittent; respiration 26; ausculation reveals the loudest mitral regurgitant murmur that I have ever heard; it could be heard at a distance of 6 inches from the thoracic parietes; auscultation and percussion yielded no other abnormal sounds. Her previous health has been excellent, and she is now quite robust for one of her extreme age; her faculties and special senses are almost unimpared. She is rather an excessive smoker, and of late has been smoking a lot of very strong tobacco. R.—Aromat. sp. ammonia; camph. tinct. opii, aa dr ii; Sul. ether, dr i.—M. Sig. Take one teaspoonful every 4 or 6 hours in some water; ordered a liberal diet.

May 8.—Symptoms very little changed, except the attacks of difficult breathing are not so frequent, and are very readily set aside by the ether mixture. Continue treatment.

May 10th.—Patient much improved; appetite good; can lie in any position without trouble of breathing; has had no attack of difficult breathing since my last visit; cardiac murmur less distinct, but still plainly audible with ear resting on chest; still complains of weakness. Continue other mixture *pro re nata*, and take following pills: R.—Iron by hydrogen, dr i; Ext. nux vomica (al.); Pulv. digitalis, aa grs xv; Paste (tragacanth and glycerine), q. s.—M. Ft. pills No. 30. Sig. One to be taken 3 times a day.

May 13th and 25th.—Patient now complains of nothing but some weakness; but the cardiac murmur is still audible; pulse very slightly intermittent; appetite is good, and she thinks that she is nearly well. Continue treatment and diet.

June 12th.—Patient entirely recovered, objectively; but the murmur is still very slightly audible; pulse almost entirely regular. She says that she is as well as she ever was in her life. R.—Ext. nux vomica, (al.); Pulv. digitalis, aa, scru. i; water q. s. M. Ft. pills No. 40. Sig. One to be taken every night and morning. Discharged.

The cause of the cardiac trouble was, doubtless, in this case, composite; the remote cause being extreme age, excessively warm weather, and excess in smoking very strong tobacco; the exciting cause being want of tension in both the parietes and fleshy columns, with possibly some organic change in the valves themselves, as ossification or calcification, or atrophy, or some other change incident to extreme old age. I have no doubt but that the murmur has entirely left before this date; and a deaf doctor, or one too lazy to make a physical examination, would have pronounced her perfectly well at my last visit.

